# Improving the management of multimorbidity in general practice: protocol of a cluster randomised controlled trial (The 3D Study)

**DOI:** 10.1136/bmjopen-2016-011261

**Published:** 2016-04-25

**Authors:** Mei-See Man, Katherine Chaplin, Cindy Mann, Peter Bower, Sara Brookes, Bridie Fitzpatrick, Bruce Guthrie, Alison Shaw, Sandra Hollinghurst, Stewart Mercer, Imran Rafi, Joanna Thorn, Chris Salisbury

**Affiliations:** 1 Centre for Academic Primary Care, School of Social and Community Medicine, University of Bristol, Bristol, UK; 2 Centre for Primary Care, Institute of Population Health, University of Manchester, Manchester, UK; 3 Institute of Health and Wellbeing, College of Medical, Veterinary and Life Sciences, University of Glasgow, Glasgow, UK; 4 Quality, Safety and Informatics Research Group, University of Dundee, Dundee, UK; 5 Clinical Innovation and Research, Royal College of General Practitioners, London, UK

**Keywords:** Family Practice, Chronic disease, Multimorbidity, Patient centred care, co-morbidity

## Abstract

**Introduction:**

An increasing number of people are living with multimorbidity. The evidence base for how best to manage these patients is weak. Current clinical guidelines generally focus on single conditions, which may not reflect the needs of patients with multimorbidity. The aim of the 3D study is to develop, implement and evaluate an intervention to improve the management of patients with multimorbidity in general practice.

**Methods and analysis:**

This is a pragmatic two-arm cluster randomised controlled trial. 32 general practices around Bristol, Greater Manchester and Glasgow will be randomised to receive either the ‘3D intervention’ or usual care. 3D is a complex intervention including components affecting practice organisation, the conduct of patient reviews, integration with secondary care and measures to promote change in practice organisation. Changes include improving continuity of care and replacing reviews of each disease with patient-centred reviews with a focus on patients' quality of life, mental health and polypharmacy. We aim to recruit 1383 patients who have 3 or more chronic conditions. This provides 90% power at 5% significance level to detect an effect size of 0.27 SDs in the primary outcome, which is health-related quality of life at 15 months using the EQ-5D-5L. Secondary outcome measures assess patient centredness, illness burden and treatment burden. The primary analysis will be a multilevel regression model adjusted for baseline, stratification/minimisation, clustering and important co-variables. Nested process evaluation will assess implementation, mechanisms of effectiveness and interaction of the intervention with local context. Economic analysis of cost-consequences and cost-effectiveness will be based on quality-adjusted life years.

**Ethics and dissemination:**

This study has approval from South-West (Frenchay) National Health Service (NHS) Research Ethics Committee (14/SW/0011). Findings will be disseminated via final report, peer-reviewed publications and guidance to healthcare professionals, commissioners and policymakers.

**Trial registration number:**

ISRCTN06180958; Pre-results.

**Strengths and limitations of this study**

- This large trial design draws on considerable evidence about problems experienced by patients with multimorbidity and is based on an evidence-based conceptual framework for how best to improve their management in general practice.
- The healthcare landscape is constantly changing and ‘usual care’ is variable; therefore, a nested process evaluation will explore how, why and in what contexts the intervention is or is not effective.
- This study is limited by its focus on how the UK National Health Service organises general practice in England and Scotland. The findings may not all be generalisable to countries which have different types of healthcare system.
- Given the lack of a universally agreed definition of multimorbidity, we have defined our multimorbidity study population based on having three or more conditions included in the UK Quality and Outcomes Framework. Although this will include participants with a wide range of disease combinations, different definitions of multimorbidity would lead to inclusion of patients with different characteristics.

## Introduction

An increasing number of people are living with multiple chronic conditions or multimorbidity. At least 16% of adult patients in primary care in the UK have multimorbidity and prevalence increases with age.^1^ ^2^ These patients experience a high level of ‘illness burden’ due to poor quality of life, high rates of depression (which often goes unrecognised) and reduced life expectancy.^2^ ^3^ They also experience ‘treatment burden’ due to having to attend multiple specialist clinics and seeing many different professionals, which can be inconvenient for patients as well as inefficient for the health service.^4–6^ They may have to take multiple medications in complex regimes.^7^ This polypharmacy can be burdensome for patients, increases the likelihood of interactions and adverse effects (including those causing hospital admissions), and may reduce medication adherence.^8–11^

In qualitative studies, patients with multimorbidity describe a lack of holistic patient-centred care, and a concern that no single professional takes overall responsibility for their treatment and treats them as a whole person.^4^ ^5^ Current treatment guidelines and professional incentive schemes tend to be focused on individual diseases, which can lead clinicians to focus on disease-based metrics rather than on the problems that are of most concern to the individual with multimorbidity.^12^ Many different sets of guidelines can be relevant to one patient with multimorbidity, and attempting to follow all of these guidelines may be excessively burdensome, inefficient and ineffective.^7^

Multimorbidity represents a challenge to healthcare systems as well as to individual patients. Patients with multimorbidity have high rates of primary care consultations and hospital admissions and they account for a disproportionate amount of overall health service expenditure.^13^ In the USA, it is estimated that 75% of the healthcare expenditure is spent on treating chronic conditions, while in Europe, the aggregated healthcare cost multiplies with each additional condition (mean cost estimate for three conditions=€1631 compared with €562 for zero conditions).^13^ From the healthcare professional's point of view, patients with multimorbidity can be challenging to manage.^14^ ^15^ Clinicians express frustration with the lack of time, fragmentation of the healthcare system and inadequate guidelines which limit the care they can offer these patients.^16^ Complex medication management is also cited as a particular issue in multimorbidity.^14–16^

The majority of healthcare for people with chronic conditions is provided in primary care, and therefore this should be the main setting for approaches to improve the management of multimorbidity. A recent Cochrane review highlighted the paucity of research on interventions to improve the outcomes of patients with multimorbidity in primary care.^17^ Ten studies were identified examining a range of complex interventions which demonstrated mixed effects. The most effective were organisational interventions focused on areas of concern for patients or where they have difficulties, such as functional ability and medication management. No studies included an economic analysis of cost-effectiveness, although a trend towards improved prescribing and medication adherence suggests the potential for cost-savings. The authors of the systematic review called for further pragmatic studies based in primary care settings, using clear definitions of participants and appropriate outcomes.

In summary, patients with multimorbidity experience problems of illness burden (poor quality of life, depression), treatment burden (multiple uncoordinated appointments, polypharmacy) and lack of person-centred care (low continuity, little attention paid to patients' priorities). This research is designed to test the hypothesis that a patient-centred intervention in general practice designed to address the needs and priorities of patients with multimorbidity will improve their health-related quality of life, reduce their burden of illness and treatment and improve their experience of care, while being more cost-effective than conventional service models. This will be examined using a cluster randomised controlled trial (RCT), with economic evaluation and mixed-methods process evaluation.

## Methods and analysis

### Trial design

**Figure 1:**
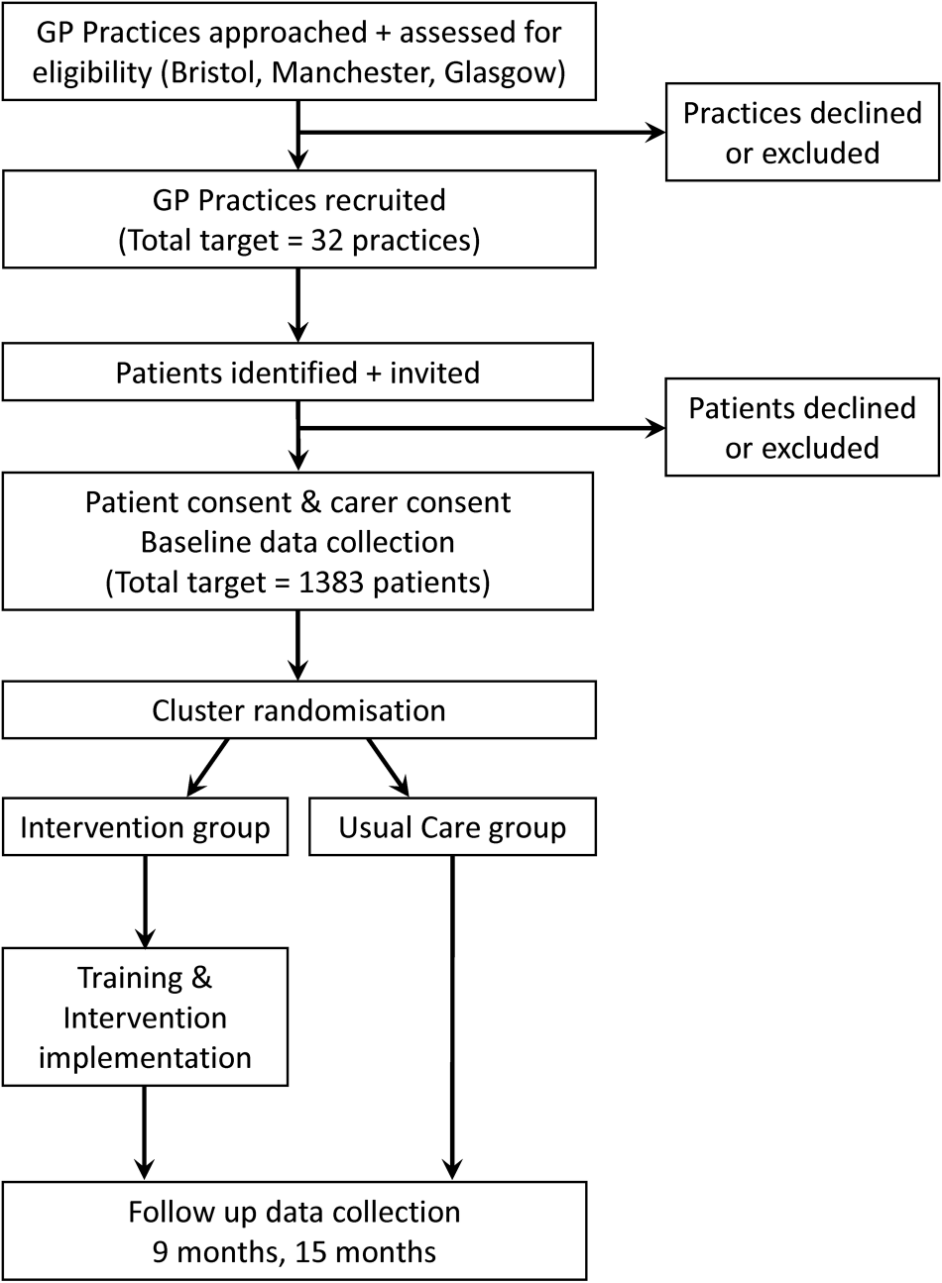
Flow chart of practice and patient recruitment, implementation and follow-up. GP, general practitioner.

This is a multicentre pragmatic, two-arm, practice-level cluster RCT (see figure 1), with parallel mixed-methods process evaluation and economic analysis of cost-effectiveness. The design is based on the Medical Research Council (MRC) framework for the evaluation of complex interventions.^18^

### Conceptual framework

The underlying theoretical basis for the intervention is the patient-centred care model.^19–21^ This includes four key components, all of which are highly relevant to improving care for patients with multimorbidity:

- A focus on the patient's *individual disease and illness experience*: exploring the main reasons for their visit, their concerns and need for information.
- A *biopsychosocial perspective*: seeking an integrated understanding of the whole person, including their emotional needs and life issues.
- Finding *common ground* on what the problem is and mutually agreeing management plans.
- Enhancing the *continuing relationship* between the patient and doctor (the therapeutic alliance).

The intervention design is based on a conceptual framework which delineates the main problems experienced by patients with multimorbidity (drawing on the existing research evidence) and uses strategies based on the patient-centred care model to seek to address these problems. The general approach has many commonalities with well-recognised frameworks such as the chronic care model^22^ and the House of Care.^23^

### Participants and setting

This study is based in general practices serving different patient populations in three geographical areas; in and around Bristol, Greater Manchester and Glasgow. Practices in this study will be selected from areas with a range of socioeconomic characteristics, particularly levels of deprivation.

In the UK, each patient is registered with one general practice, typically with between 2 and 10 general practitioners (GPs) and a smaller number of practice nurses. Patients receive almost all of their primary medical care from their general practice, which acts as gatekeeper to secondary care services. Patients with multimorbidity are called in for regular review of each of their medical conditions, often having separate reviews for each condition. Many reviews are conducted by nurses who use disease-specific computerised templates to collect relevant data according to clinical guidelines.

### Inclusion and exclusion criteria

#### General practices

To be eligible for inclusion practices need a minimum of three GP partners, a minimum list of 4500 registered patients and to use EMIS Web or EMIS PCS as their computer system. EMIS is the most common clinical records system in UK general practice.

#### Patients

Inclusion criteria are being aged 18 years or over, being registered with a usual doctor who is participating in the research study and having three of more chronic conditions from those included in the National Health Service (NHS) Quality and Outcomes Framework^24^ (QOF, V.31.0)—see box 1.

Box 1 **Chronic conditions for inclusion**

Included patients have three or more diagnoses from the following groups of chronic conditions:

- Cardiovascular disease or chronic kidney disease (including coronary heart disease, hypertension, heart failure, peripheral arterial disease, chronic kidney disease stage 3–5)*
- Stroke
- Diabetes
- Chronic obstructive pulmonary disease or asthma*
- Epilepsy
- Atrial fibrillation
- Severe mental health problems (schizophrenia or psychotic illness)*
- Depression
- Dementia
- Learning disability
- Rheumatoid arthritis

*Groups are counted only once even if a patient has multiple conditions within a group. For example, having both hypertension and heart failure would just count for one condition.

Exclusion criteria are: having a life expectancy of less than 12 months; serious suicidal risk; known to be leaving the practice within 12 months; unable to complete questionnaires in English even with the help of carers; actively taking part in other research involving extra visits to primary care or other health services; lacking capacity to consent (as coded in their practice records, or determined by their GPs, in Scotland only); being considered unsuitable for the research study by their GP (eg, recently bereaved or currently hospitalised).

### Carers

Formal or informal carers of patients consenting to take part in the study will also be invited to contribute by completing a carer's questionnaire. Not all patients may have carers and not all carers may want to take part; therefore, this constitutes a small and separate substudy population.

### Recruitment of practices

General practices which are potentially interested in taking part in the trial will be identified with help from the NHS Clinical Research Networks in England and the Scottish Primary Care Network. These nationwide networks facilitate clinical research by identifying and recruiting general practices and providing resources to help practices do research. Local researchers will meet with key stakeholders at the practice (practice manager, GPs, practice nurses) in order to explain the study and its requirement of a commitment to organisational and procedural change. The practice manager or lead GP will sign a practice-level consent agreement.

### Recruitment of patients

Each participating practice will be asked to search their practice database using a standard electronic search provided by the research team to identify potentially eligible patients who have three or more chronic conditions as defined by the inclusion criteria. In some practices, not all GPs will participate, so in practices which have a policy for patients to see the same GP, only those patients who usually see one of the participating GPs will be included. This is to minimise the potential distress of asking a patient to change their GP for the purpose of the study. If there are more than 150 eligible patients, a simple random sample of 150 of these patients will be selected. GPs will be asked to review the resulting list to screen out patients meeting the exclusion criteria. The practice will send the remaining patients a patient invitation pack including information about the study (see online supplementary appendix 1), a consent form (see online supplementary appendix 2) and baseline questionnaire. Non-respondents will be sent one postal reminder, supplemented by a telephone reminder when possible in practices where recruitment targets are not met.

10.1136/bmjopen-2016-011261.supp1 Supplementary data

10.1136/bmjopen-2016-011261.supp2 Supplementary data

At sites in England, if a patient lacks capacity to consent, we will obtain the assent of the patient's carer, legal guardian or consultee on behalf of the patient to take part in the study. Carers will be invited to complete a separate carer contact form, and those who wish to participate in the carer's substudy will be sent an information sheet, consent form and baseline questionnaire.

Recruitment of patients began on 20 May 2015 and ended in December 2015. Intervention training began in June 2015 with intervention delivery period starting in August 2015 and due to finish in March 2017.

### The intervention

#### Development

The intervention was developed to address the problems identified in earlier qualitative and quantitative research on the problems experienced by patients with multimorbidity,^25–27^ along with experience from previous trials summarised in a systematic review.^17^ This was followed by a series of workshops and stakeholder events with patients, carers, health professionals and health service managers. This resulted in a complex intervention with multiple interacting components at the different levels of individual patient–clinician interactions, practice organisation, primary–secondary care integration, and measures to support and incentivise practices to make changes in their services.

Three general practices participated in an external pilot and feasibility study in which the feasibility of the intervention was assessed and improved, and aspects of trial delivery were tested. The views of the patients and healthcare professionals delivering the intervention were fed back to the research team. The key learning points and changes resulting from the optimisation phase are described in online supplementary appendix 3.

10.1136/bmjopen-2016-011261.supp3 Supplementary data

#### Intervention components

**Figure 2:**
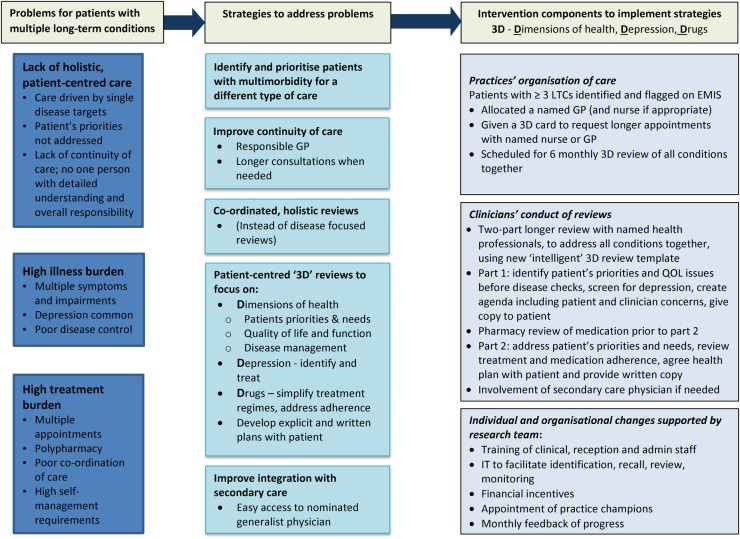
3D logic model. GP, general practitioner; LTC, long term conditions; QOL, quality of life.

The name ‘3D’ was chosen because it acts as a mnemonic for ‘dimensions of health; drugs; depression’ and also because it alludes to the concept of a holistic, three dimensional perspective. The main components of the final 3D intervention, to be tested in the definitive trial, are illustrated in figure 2 and described below.

The problems experienced by patients with multimorbidity in current care were broadly grouped under the headings of a lack of holistic patient-centred care, high illness burden and high treatment burden. Strategies were identified to try to address each of these problems, as shown in the middle column of figure 2. Finally the specific operational mechanisms or active components of the intervention which will be used to implement each strategy are described in the third column.

#### Components at practice level relating to organisation of care

The aim is to identify a group of patients with high levels of multimorbidity and on several QOF disease registers in order to prioritise them for a different form of care, recognising that they have more complex needs than most patients. Consenting patients with multimorbidity (as defined in box 1) will be identified and ‘flagged’ on practice computer systems. They will be allocated a named GP with responsibility for their care (and nurse if possible, particularly in larger practices where several nurses are involved in chronic disease management). These patients will be provided with a ‘3D’ card in order to identify themselves with practice receptionists when booking appointments. The 3D card reminds the patient of their named responsible GP, and encourages them to ask for a longer appointment than usual when they think they need one. This recognises that these patients often need to discuss several problems at one appointment.

In most general practices in the UK, patients with one of the chronic conditions listed in box 1 are invited for review of that condition on a regular basis, such as every 6 or 12 months. At these reviews, the GP or nurse follow computerised disease management templates to collect relevant data about aspects of disease control and management. Patients with multimorbidity may be repeatedly called for separate reviews of each of their chronic conditions, often to see different health professionals, who use different disease management templates which include a large amount of duplication (eg, most templates include measurement of blood pressure and asking about smoking habits). Under the 3D approach, these separate disease-focused reviews will be replaced by a 3D review every 6 months at which all problems will be reviewed at one time.

#### Components relating to clinicians conduct of reviews

The 3D reviews are comprehensive and, although they include the important aspects of disease management included in single disease reviews, they have a different focus. The conduct of the reviews will be supported by a bespoke ‘dynamic’ template which automatically personalises for individual patients to only include prompts relevant to the conditions that the patient is recorded as having in the electronic medical record. It eliminates the problem of duplication of information between different single disease templates and also provides a structure to encourage the clinicians to enact the 3D approach. The term ‘3D’ acts as a mnemonic to encourage clinicians to focus on the following:

- Dimensions of health: This includes first eliciting patients' concerns and priorities for improving their quality of life and function, before collecting data about disease metrics such as weight or blood pressure.
- Depression: The clinicians should screen for depression and seek to treat it if identified.
- Drugs: In order to address problems of polypharmacy, a pharmacist will review the patient's medical records prior to the 3D review and make recommendations about low priority drugs that might be discontinued, or other ways of simplifying drug regimes, for example, using long-acting medications, so that all tablets can be taken in the morning. The pharmacist review is performed online through remote access to patients' electronic medical records by prior arrangement between the practice and pharmacist. As part of the 3D review, the GP will be trained to ask questions to detect problems with medication adherence and how to help the patient to address this.

Each six-monthly 3D review consists of two appointments. At the first appointment (lasting approximately 30–40 min), the practice nurse will collect information to complete the template and organise all relevant blood tests or other investigations. The nurse review includes collecting information about the patient's priorities for change and aspects of quality of life such as pain and function, and also includes screening for depression using the Patient Health Questionnaire 9 (PHQ9) questionnaire.^28^ Following the nurse appointment, the patient will be given a document, known as the ‘3D agenda’, which summarises their assessment and details their top priorities for change. This will set the agenda for the second appointment, approximately 1 week later, with the patient's usual GP. At this 20 min appointment the GP will review all the information collected by the nurse and from the test results, undertake a thorough medication review with the help of the pharmacist's recommendations, seek to address the patient's priorities and problems identified in the assessment, and agree a written care plan for the patient to take away. This 3D Health Plan spells out the specific problems identified (which may be a combination of patients' priorities and problems identified by the nurse or doctor during the review), along with mutually agreed actions that patients and clinicians each have responsibility for. Providing patients with a 3D health plan as a printed summary of their 3D review and test results is intended to promote patient engagement.

Each general practice will be allocated a designated ‘generalist physician’ (usually a geriatrician) in secondary care whom they can contact to discuss individuals with complex problems and (if possible) to help coordinate use of hospital investigations and appointments where patients are attending numerous different specialist clinics or having multiple hospital-based tests on different days.

#### Components relating to supporting practices

In order to support the implementation of the intervention, the study team developed a training package of two half-day sessions for healthcare professionals. These sessions are facilitated by a clinician trainer and at least one local researcher, covering tasks and discussion topics including eliciting of patient concerns, exploring strategies to promote patient-centred care, ways to improve continuity of care, negotiating a patient health plan, improving medication adherence, the aims of the 3D reviews and use of the 3D review template. A substantial element of the training will be devoted to promoting attitudinal change among clinicians towards identifying and responding to patients' own priorities and problems with broader quality of life, as organisational change is unlikely to be effective unless clinicians ‘buy into’ the underlying philosophy of the new approach.^29^ Practice receptionists will also be offered training in promoting continuity of care and offering longer appointments to patients with multimorbidity.

A number of other strategies are being followed to promote implementation of the intervention within practices. In addition to the bespoke computerised 3D review template, we have also developed software to facilitate identification and monitoring of the participants. Financial reimbursement is provided to practices to cover the costs of practice staff training and setting up of the necessary patient recall systems. Modest financial incentives (£60 per patient) are also provided to practices based on the number of patients that complete both of their six-monthly 3D reviews within the 15-month follow-up period. Each practice will be asked to nominate a GP champion to help monitor and promote the intervention within the practice, and also to meet and share good ideas and experiences with other GP champions in local collaboratives. The practice champions will be provided with monthly feedback reports about their practice's progress in implementing the reviews. We will allow local adaptation of the intervention to reflect local context while ensuring the key elements of the conceptual framework (those shown in figure 2) are maintained.^30^

### Control group

Patients in practices allocated to the control arm will continue to receive care as usual. In most practices, this will mean patients are recalled to different clinics to see different practice nurses to review each of their long-term conditions. The nurses will usually follow disease-specific computerised protocols for their management, and will mainly focus on collecting data related to QOF targets rather than quality of life or patients' priorities. The nature of ‘care as usual’ may vary between practices and over time—this will be explored in the process evaluation.

### Participant withdrawal

Among intervention practices, if any participant later requests not to receive the 3D intervention, they will revert to the usual care provided for other patients in their practice. Unless a patient requests to withdraw from the trial they will continue to be followed up and will be analysed in the group to which the practice was allocated. If they wish to withdraw from the trial, then no further follow-up data will be requested but data already provided will be used.

### Outcome measures

#### Primary outcome measures

The primary outcome for patient and carers will be health-related quality of life (HRQoL) as measured by the EQ-5D-5L after 15 months following patient recruitment.^31^ The EQ-5D is a widely used self-reported generic measure of HRQoL which has been validated in many different patient populations including diabetes, cardiovascular problems, chronic obstructive pulmonary disease, cancer, chronic pain and rheumatoid arthritis. The five-level version (EQ-5D-5L) contains the same dimensions as the earlier three-level version (EQ-5D-3L) but has been designed to provide greater reliability and sensitivity.

#### Secondary outcomes

Secondary outcome measures for participants are grouped under domains as shown in box 2.

Box 2 **Secondary outcomes for patients participating in the 3D trial**

*Experience of holistic patient-centred care*

- Consultation and Relational Empathy (CARE) measure of relational continuity in general practitioner and nurse* consulations^32^
- Coordination of care (two questions from LTC6 Quality Innovation Productivity and Prevention (QIPP) programme)
- Patient Assessment of Chronic Illness Care (PACIC) measure^33^
- Overall satisfaction (single item)

*Burden of illness measures*

- Self-rated health
- Illness burden in multimorbidity (Bayliss)^34^
- Quality of disease management (a composite measure of Quality and Outcomes Framework (QOF) achievement)^35^
- Hospital Anxiety Depression Scale (HADS)^36^

*Burden of treatment*

- Brief Treatment Burden Questionnaire†
- Morisky Medication Adherence Scale (eight-item)^37^
- Number of prescribed drugs
- Number of high-risk drug combinations^38^

*Not collected at 9 months follow-up

†New measure developed for this study, based on qualitative interviews, item generation, principle components analysis and testing of psychometric properties.

Secondary outcome measures for carers will assess measures of carer quality of life and strain, including the EQ-5D-5L,^31^ the Carer Experience Scale^39^ and the Brief Treatment Burden Questionnaire for carers. These will be reported separately, as they are not participant outcomes.

#### Measures of process of care

We will monitor processes in the intervention practices in order to report the degree of implementation of the intervention. This will include the number of nurse and GP 3D reviews undertaken, the extent to which the 3D template was fully completed, the number of pharmacy reviews performed, whether an agenda and health plan were created and printed off to give to the patient, and the number of times the hospital general physician was contacted.

The continuity of care (COC) measure^40^ will be used as a measure of longitudinal continuity, for all telephone or face-to-face consultations by participants with GPs or nurses within the practice over the 15-month follow-up period, adjusted for continuity in the 15 months before the intervention.

Although also required for the economic analysis, the number of primary care consultations and the number of hospital admissions will be of particular interest as indicators of the effect of the intervention on primary and secondary health services.

We will report descriptively the systems in place to provide care for patients with multimorbidity in practices in both arms of the trial at baseline and at the end of the 15-month follow-up period, in particular to capture whether there are differences in ‘usual care’ in the control arm practices over the period of this study.

### Economic evaluation

The economic evaluation will be undertaken from the perspectives of (1) NHS and personal social services (PSS) and (2) patients. We will compare the extra cost of caring for patients in the intervention group with the difference in outcome as measured by the EQ-5D-5L and related quality-adjusted life years (QALYs). Resource use data will be collected from patient self-reported postal questionnaires at baseline, 9 and 15 months and GP practice records. The questionnaires will ask about the use of community and secondary care health services, social services, informal care, and personal costs (including travel, loss of earnings and dependent care costs). Patients indicating use of hospital services will be contacted by telephone to obtain more detail about the inpatient stay or accident and emergency visit. GP practice records will be used to obtain information about all available primary care contacts, including type of consultation and who was seen, tests and investigations, and prescribed medication.

Trial records will be used to estimate the cost of setting up the 3D service and training staff. This will be identified and reported separately from the running costs.

NHS resources will be valued using national published sources such as Curtis,^41^ NHS reference costs^42^ and the British National Formulary (BNF).^43^

### Data collection

At baseline, data will be collected on the sociodemographic measures (number of long-term conditions; age; gender; education; ethnicity; deprivation status (index of multiple deprivation based on postcode); work status) and all primary and secondary outcomes. The primary outcome will be collected 9 and 15 months after recruitment, with the primary outcome time point being at 15 months. All but one of the secondary outcomes will be collected at 9 months, as shown in box 2. All secondary outcomes, measures of the process of care and measures of resource utilisation will be collected 15 months after recruitment. Practice randomisation occurs after patient recruitment, and it then takes approximately 3 months to train practices to deliver the 3D intervention. Patients have their 3D reviews on a six-monthly cycle. Therefore, collecting outcome data 9 and 15 months after patient recruitment allows for a 3-month lag time and ensures that most patients will be invited to have two 3D reviews before outcomes are measured.

The primary method of self-reported data collection will be via postal questionnaires; however, alternative completion methods including by telephone or via a home visit by a researcher masked to treatment allocation will be offered if necessary in order to maximise response rates.

Two reminders, the first by letter or email (approximately 10–14 days after posting the questionnaire) and the second by phone (approximately 10–14 days after the first reminder), will be made for participants who have not returned their questionnaire. Patients will be given £5 gift vouchers for completion of questionnaires.

No data about identifiable patients will leave the practice unless patients have provided consent. All data will be stored securely and confidentially at the University of Bristol in line with its data management policies.

### Sample size

The study is designed to detect an effect size of 0.274 SDs in the primary outcome of the EQ-5D-5L. Data about the variability of the new five-level (5L) version of the EQ-5D is currently more limited than for the well-established three-level (3L) version. The SD of the EQ-5D-3L in the UK general population is 0.23, rising to 0.27 in the oldest respondents (aged over 75).^44^ Hence, an effect size of 0.274 would equate to a detectable difference of (0.274×0.27)=0.074 on the EQ-5D-3L, previously deemed to be the minimum important difference.^45^ Although there are less data about the variability in the 5L version of the EQ-5D than the 3L version, this latest version is likely to have greater sensitivity to change.^31^

Based on data available from our previous studies,^1^ we estimated that 2.3% of adult patients would have multimorbidity as defined in this study. This equates to about 108 patients in an average-sized practice of 6000 patients. Recruiting 32 practices would therefore provide 3456 potentially eligible patients. Assuming 40% of patients agree to participate (n=1382), 80% are followed up to 12 months, and an intraclass correlation coefficient (ICC) of 0.03 for clustering at the practice level (based on the WISE trial),^46^ 32 practices will provide approximately 90% power, with a 5% α level to detect an effect size of 0.274 SDs in the EQ-5D-5L measure between the intervention and control groups.

### Allocation

General practices will be the unit of allocation. Practices will be allocated in a 1:1 ratio to receive either the intervention or continue care as usual (control group). Randomisation will be stratified by area (Bristol, Greater Manchester, Glasgow) and minimised by deprivation level and practice size. Within each area allocation will be performed in blocks of two, with both practices in a block randomised at the same time and released to the trial manager together to ensure allocation concealment and no selection bias. It was not deemed possible to increase or vary the block sizes given the small number of practices recruited to each area and the dynamic nature of recruitment. The trial manager will notify the local research team of the two allocations and they will then notify the practices and arrange training of the intervention practice. The allocation schedule will be computer-generated by the trial statistician, blind to details of the practices apart from those needed for stratification and minimisation.

Randomisation of a practice will take place after patients in that practice have been identified and invited to participate in order to avoid selection bias.

### Blinding

Once participants have been recruited, it will not be possible to mask participants or healthcare professionals to the group allocation of their practice. It is also not feasible to blind all members of the study team actively involved in the execution of the study. However, data entry and checks of data quality will be conducted by administrative staff masked to treatment allocation. Analysis of outcomes will be performed by the trial statistician, also masked to treatment allocation.

### Statistical methods

Data will be analysed in accordance with CONSORT principles and its extension for cluster randomised trials. Descriptive statistics will be used to summarise characteristics of practices and patients and compare baseline characteristics between groups. A full statistical analysis plan will be developed and agreed by the Data Monitoring Committee (DMC) and the Trial Steering Committee (TSC) after completion of the pilot phase and prior to undertaking any analyses of the main trial.

All analyses of primary and secondary outcomes will be at the patient level and will account for clustering by practice using multilevel regression models. Analyses will be performed on an ‘as allocated’ basis. Primary analysis comparing EQ-5D-5L between the intervention and control practices will employ a linear multilevel regression model adjusted for stratification/minimisation variables. Subsequent models will adjust for baseline EQ-5D-5L, any variables demonstrating imbalance at baseline and other important prognostic variables such as age, number of long-term conditions, deprivation and depression. Preplanned analyses of secondary outcomes will also employ linear or logistic (as appropriate) multilevel regression models.

Formal tests of interaction will be performed to consider the following potential effect modifiers: age, number of chronic conditions, index of deprivation, and presence or absence of depression alongside physical health problems. The trial is not specifically powered for such interaction tests; hence, interpretation will focus on the CIs and will be hypothesis-generating only. The potential impact of missing data will be examined through sensitivity analyses.

Anonymised data will be used in order to compare descriptive data for consenting versus non-consenting patients. We will explore the possibility of comparing QOF performance in patients with chronic conditions both with and without multimorbidity—this is to assess for the potential unintended consequence that concentrating effort on patients with multimorbidity may have a positive or negative impact on the care of other patients.

No interim analyses are planned.

### Economic analysis

Cost per patient will be estimated by applying unit costs to the resources used. In a cost-consequences analysis, we will relate the mean cost per participant in each group with changes in a range of outcomes; cost-effectiveness analysis from the NHS and PSS perspective will estimate the incremental cost per QALY gain where QALYs are estimated using the EQ-5D-5L. Uncertainty will be addressed in sensitivity analyses and by using bootstrapping to estimate the net monetary benefit and a cost-effectiveness acceptability curve.

### Process evaluation

Alongside the main analysis of quantitative outcomes from the trial, we are conducting a nested process evaluation. This mixed-methods study aims to better understand how and why the intervention was effective or ineffective and to identify contextually relevant strategies for successful implementation as well as practice difficulties in adoption, delivery and maintenance of the intervention. Further details of the protocol for the process evaluation will be published in a separate paper.^47^

## Ethics

### Ethics approval

This study will be conducted in accordance with principles of good clinical practice.

Patients will not be denied any form of care that is currently available in the NHS by participating in this study. Patients from usual care practices will still have access to all locally recommended treatments and services. Patients from intervention practices will still have full access to their GP and secondary care services in addition to their six-monthly 3D assessments. Any changes in medication prescribing will be performed by a GP in the context of normal clinical care.

### Patient safety

We will monitor and report descriptively the numbers of serious adverse events in each arm which appeared to be related to the intervention or the trial, and also the number of deaths in each trial arm. Given that patients with multimorbidity may be heavy users of secondary care services, new medical diagnoses, hospital admissions and deaths are expected and will not be considered as potential serious adverse events unless anyone involved in the study (participants, general practice staff or research staff) notify the research team of any events that they consider may have been related to the intervention or the research process. All deaths will be investigated for relatedness by requesting the patient's GP provide details of cause of death and relatedness to study.

### Study management and oversight

The 3D Study is managed by the Trial Management Group, consisting of the chief investigator, principal investigators and researchers from each of the recruiting sites and other co-applicants. There is additional governance oversight by an independent TSC and an independent DMC, both constituted in line with guidance from the National Institute for Health Research (NIHR). An advisory group with members from key local and national stakeholder organisations and lay members has been convened to provide advice about the wider context, other related initiatives and to facilitate communication and eventual knowledge mobilisation with regard to this trial. There is an active patient and carer forum which meets regularly to advise on the design and conduct of the study.

The project will seek to maximise the impact of the research by adopting a model of knowledge transfer. We aim to disseminate our findings to patients, healthcare professionals, commissioners and other academics. In addition to publication of study results, guides for commissioners and for practices will be produced to enable wider implementation of the new 3D approach. The RCGP Clinical Innovation and Research Centre will facilitate wide dissemination to practices and the production of these resources.

The research team is committed to full publication of the results. Authorship will be in accordance with the guidance of the International Committee of Medical Journal Editors. All authors will have full access to the study data. Once the main results have been published, data may be available to other investigators subject to agreement about the protocol with the chief investigator and compliance with policies of the funder and sponsor in relation to data sharing. The study sponsor and the funder will have no role in study design, data collection, management, analysis or interpretation of data, writing of the final report or the decision to submit for publication.

## Discussion

This large and rigorous trial will provide robust evidence about the benefits and costs of a pragmatic intervention to improve the management of multimorbidity in general practice. It builds on a considerable evidence base about the difficulties experienced by patients with multimorbidity and the health professionals who seek to care for them. Through the use of a patient-centred conceptual framework, it tests a range of strategies which should address these difficulties and improve outcomes that matter to patients. The study is highly pragmatic.^48^ It is based in a range of normal general practice settings and in the different health economies of England and Scotland, which will enhance generalisability. It includes patients with broad inclusion criteria and few exclusion criteria, and assesses a wide range of outcomes including those relating to health status, patient experience and resource utilisation. Implementation of the intervention is flexible to local context, but the extent to which the intervention adheres to the key-intended principles will be monitored.

The study is being conducted with considerable attention to principles of knowledge translation. If the intervention is effective, it will be possible to roll it out quickly to general practices across the UK, and the 3D approach is also likely to be applicable to the management of patients with multimorbidity in many other countries.
