# Supplementary material for: Improving the management of multimorbidity in general practice: protocol of a cluster randomised controlled trial (The 3D Study)
Source: BMJ Open. 2016 Apr 25;6(4):e011261. doi: 10.1136/bmjopen-2016-011261 (PMC4854003; doi:10.1136/bmjopen-2016-011261)
Supplement: Supplementary data [file bmjopen-2016-011261supp1.pdf]

The 3D Study:

Improving whole person care

Patient and Carer Information Booklet

We invite you to take part in a research study

- Thank-you for reading about the study. We would like to explain why it is being done.
- Before you decide whether to take part, please take time to read this booklet carefully and talk to others if you wish so that you are sure that you understand what it would involve for you.
- If you are a carer/guardian or responsible for someone with several health problems they can still take part in the study. We would be interested in your views as a carer. Please complete the carer contact form or contact the research team to discuss what this involves and we will send you separate information and a form to complete.

How to contact us

If you have any questions about this study, please contact:

<Local Researcher Name>
<Local Research Site Address1>
<Local Research Site Address2>
<Local Research Site Address3>

Telephone: <Local Researcher phone number>
Email: <Local Research site email address>

Purpose of the study

The aim of this study is to help GP surgeries to manage and support people who have several long-term health problems, such as diabetes, asthma, heart disease or arthritis.

People who have several health problems often have lots of appointments, see lots of different doctors and nurses and have to take lots of different tablets.

In this study, researchers will work with half of the GP surgeries that are taking part in the study to change how they provide care for this group of patients. These practices will be in the **'New Approach' group.** These changes include arranging a review of all your conditions together every 6 months, trying to make it easier for patients to see the same doctor, offering longer appointments when necessary, trying to simplify the number of drugs patients have to take, and giving patients their own 'health plan'.

The other half of the GP surgeries that are taking part in the study will continue to provide care in the same way as usual. These practices will be in the **'Usual Care' group.**

Practices will be put into one of these two groups by chance, like tossing a coin.

We will follow up patients who agree to help with the study in both groups of practices for 1 year. We are doing this study to find out what patients think about different ways of organising the service, and whether this has any effect on their health, how they feel and on their use of other NHS services.

Why have I been invited?

Your GP surgery has agreed to be part of this research. They have carried out a search of their computer records and invited about 150 people who have 3 or more long-term health problems to be involved. We hope that about 1,400 people across the UK will agree to take part.

What will happen if I take part?

Based on your records, your practice has identified you as someone who would be suitable for this study. Now we are asking whether you are willing to take part in order to find out whether the new approach is better than the usual way of providing care.

If you agree to take part, and your practice is chosen to try out the new way of organising care, you will be offered this. It will include offering you a full check-up every 6 months to review all of your problems together rather than having separate check-ups for each of your problems.

On the other hand, if your practice is chosen for the 'Usual Care' group, then the way your practice organises care for you will not change as a result of this study.

If you agree to help in this way, researchers will follow you up for 1 year. You will be asked to complete a questionnaire before starting the study (the yellow questionnaire included in this pack) and then similar questionnaires 6 and 12 months later. These questionnaires ask about your health and wellbeing, how you cope with your illnesses and what treatments and other health services you use. The research team will send you a £5 gift voucher as a token of appreciation for your help with completing each of these questionnaires.

The research team would also like your permission to see your health records to get information about the number of appointments and treatments you have in the year before and the year after you agree to take part. All information will kept in strict confidence.

What will I have to do?

If you agree to take part in this study, then please fill in the green CONSENT form and yellow QUESTIONNAIRE. Return BOTH of these to us using the pre-paid envelope provided.
We will ask you to complete a similar questionnaire 6 months and 12 months later.

Possible additional involvement

Out of the 1,400 people in this study, we will also invite about 30 people to take part in an interview or focus group about their experience and about 20 people to have one of their 3D reviews observed. If you are invited, we will contact you again later and give you more information then so that you can decide whether or not to help with this part. You do not have to decide now. On completion of an interview or focus group, you will be given a £5 gift voucher as a token of our appreciation of your help with this part of the study.

What are the choices?

Agreeing to take part means you agree to complete questionnaires and to allow researchers to see your records **in confidence**. If your practice is in the 'New Approach' group, you will be offered this new way of providing care.

If you DO NOT wish to take part, please return a blank questionnaire to us in the pre-paid envelope. You will not be contacted again about the study.

What are the benefits and risks?

If your practice is chosen for the New Approach group, you may benefit personally by having longer appointments with your named doctor or nurse to discuss what is important to you. You may prefer the convenience of having more co-ordinated checks on your health problems and you may get benefits from having a health plan and your medications simplified. You may have to change your routine about what clinics you go to and what medications you take, but you can discuss any changes in your treatment with your doctor.

If your practice is in the 'New Approach' group, your GP may ask you questions about your mood. Some people may find this uncomfortable, but these questions are asked because some people with several long-term conditions get depressed. GPs can offer treatment for this if necessary.

By completing questionnaires and consenting to researchers seeing your records in confidence, you will help in the planning of services that GP practices can offer people with several long-term health problems. This will benefit future patients.

If you consent to complete questionnaires, this will mean giving up some of your time to do this.

If you become unwell or experience any unwanted effects, you should inform your GP. If you think that this happened because you have

received the new approach, then please also inform the research team because we need to record and report any incidents like this.

What if new information becomes available?

Sometimes new information about health care comes out. If this happens, your GP will discuss with you whether your health care should change and whether your part in the study should continue.

What happens when the research stops?

Your help with this study is complete once we receive your 12 month follow-up questionnaire. You will continue with your usual GP care as before. It is up to your GP practice to decide whether they make any continuing changes to the service they provide.

The study is due to finish in 2017. All GP practices, patients and carers who have taken part will be sent a summary of the results.
The results will be published in a report for the NHS and for use by academics, health professionals and NHS managers. **It will not be possible to identify individual patients or carers in any of the reports or summaries.**

What if there is a problem?

This research follows guidelines and does not include any new treatments. It covers health care that you would normally receive, but organised in a different way.

If you are concerned about any aspect of the study, please contact one of the research team (contact details on the front of this leaflet). Or you can contact the Study Manager at the University of Bristol, by phone <phone number> or email <email address>.

If you are still unhappy and wish to make a formal complaint, you can (a) contact your GP who can advise on local NHS complaints procedures, (b) contact the <NHS Patient Advice and Liaison Service (PALS)/ customer contact centre/patient feedback service> at your <local Clinical Commissioning Group (CCG)/NHS England/Health Board>. Details can be found at <NHS choices/England/Health board website>.

What if I don't want to carry on with the study?

Helping with this study is voluntary and you are free to stop at any time without giving any reason. The information you provided up to that point would still be used. However, you can request us to remove all your information. Please contact the research team to let us know your wishes.

If you become unwell, you, your family or a person who looks after you could contact us on your behalf and ask us to stop sending you questionnaires and/or ask us not to use your records in the research. This would not affect your rights or future care in any way.

Confidentiality

Any information collected about you during the study will be treated in confidence. It will be stored securely at the University in Bristol, Manchester or Glasgow (whichever is nearest to you). Apart from the research team, the only people who can see your information are from the NHS authority whose job it is to check the conduct of research in the NHS. If we become concerned about your health or safety during the study, we will notify you and your GP.

Other Information about the study

The research is being run by the Centre of Academic Primary Care at the University of Bristol, with the Universities of Manchester and Glasgow. It is funded by the NHS National Institute for Health Research (NIHR). All research in the NHS is looked at by an independent group of people called a Research Ethics Committee, to protect your safety, rights, well-being and dignity. This study has been reviewed and approved by the South West (Frenchay) NHS Research Ethics Committee.

What do I do now?

- **If you have any questions** about the study please contact the researchers using the details on the front of this leaflet.
- **If you would like to take part**, please fill in the green 'Consent form' AND yellow 'Questionnaire' and post them to the research team in the freepost envelope provided.
- **If you prefer not to take part**, please post the blank questionnaire only to the research team in the freepost envelope provided.

THANK-YOU FOR YOUR TIME

NHS
Bristol Clinical Commissioning Group

University of BRISTOL   MANCHESTER 1824 The University of Manchester   University of Glasgow   University of DUNDEE
