# Supplementary material for: Improving the management of multimorbidity in general practice: protocol of a cluster randomised controlled trial (The 3D Study)
Source: BMJ Open. 2016 Apr 25;6(4):e011261. doi: 10.1136/bmjopen-2016-011261 (PMC4854003; doi:10.1136/bmjopen-2016-011261)
Supplement: Supplementary data [file bmjopen-2016-011261supp2.pdf]

Patient Consent form v2.0; 24/03/15
NHS/EMIS Number: «NHS/EMIS_number»
«Title» «Forename_s» «Surname»
«House_nameFlat»
«No_and_street»
«Village»,
«Town», «Postcode»

# The 3D Study:
## Improving whole person care

# PARTICIPANT CONSENT FORM

**Title of Project:** The 3D study**:** Improving whole person care

**Principal Investigator:** Prof Chris Salisbury, University of Bristol

Thank-you very much for your interest in taking part in this research. Please read and write your initials against EACH of the following statements.

| | Please Initial EACH box |
|---|---|
| 1. I confirm that I have read the information sheet <version 2.0, dated>. I have had the opportunity to consider the information, ask questions and (if applicable) have had these answered satisfactorily. | ☐ (Your Initials) |
| 2. I understand that if my practice is chosen to try the new way of providing care for people with long term health conditions this will be offered to me, and I agree to complete questionnaires about my experience now, and in 6 months and 12 months time. | ☐ (Your Initials) |
| 3. I understand that my participation is voluntary and that I am free to withdraw at any time without giving any reason and without my medical care or legal rights being affected | ☐ (Your Initials) |
| 4. I understand that, where relevant to this research project, my medical notes and data collected during the study may be looked at by members of the research team, regulatory authorities and NHS Trust. I give permission for these individuals to have access to my records as necessary. | ☐ (Your Initials) |
| 5. I agree to my GP and health care professionals being informed of my participation in the study. The research team and GP can share information about my health and health care where relevant to the research study. | ☐ (Your Initials) |
| 6. I agree that the research team can use my personal details to contact me about the study. I understand that my participation is confidential. | ☐ (Your Initials) |
| 7. **I agree to take part in the above named study** | ☐ (Your Initials) |

**Please complete the following details, and return this form & the completed BASELINE QUESTIONNAIRE to the research team in the pre-paid envelope**.

|________________________________|  ______________  ________________________

Full Name (BLOCK CAPITALS)  Today's Date  Signature

Telephone number:______________________  Mobile number:______________________________

Email address: __________________________________________  Date of birth:__________________

Contact preference: morning/afternoon/evening/don't mind/other:______________________________

GP usually seen: ________________________________________

|________________________________|  ______________  ________________________

Researcher Name (BLOCK CAPITALS)  Date  Researcher signature

To be completed by research team when received. 1 copy for participant; 1 for GP; 1 for research centre

University of BRISTOL · MANCHESTER 1824 The University of Manchester · University of Glasgow · University of Dundee · NHS Bristol Clinical Commissioning Group
