# Supplementary material for: Improving the management of multimorbidity in general practice: protocol of a cluster randomised controlled trial (The 3D Study)
Source: BMJ Open. 2016 Apr 25;6(4):e011261. doi: 10.1136/bmjopen-2016-011261 (PMC4854003; doi:10.1136/bmjopen-2016-011261)
Supplement: Supplementary data [file bmjopen-2016-011261supp3.pdf]

Appendix 3 Learning and amendment from external pilot phase

The table below shows the procedures first trialled in the pilot study, the problems revealed by the process evaluation from feedback interviews with GPs and nurses, focus groups of patients, observation of training and review appointments and discussion with the patient and public involvement (PPI) group and finally the changes that these suggestions led to which established the processes to be followed in the main 3D study.

**Pilot procedure**	**Difficulties raised**	**Changes in Main study**
Patient consent was specifically for questionnaires and notes review. All invited patients were to be offered the intervention.	Patients unclear what they were consenting to. Non-consented patients called for review and refusing the 3D reviews.	Patient information improved and patients now consenting to all trial procedures, not just questionnaire and notes review.
Practices should deliver the intervention to all invited participants (approx. 140 patients). Requiring whole practice change, training of most practice staff.	Practice concerns of workloads. Difficulty of arranging training for majority of practice staff. Disruption of existing timetabling of clinics and appointments.	Practices only delivering to consented patients (approx. 40-50 patients – seen as more achievable). Less staff required for training. Less disruption.
Mixed practice training sessions offered off-site to allow sharing of experiences.	Difficult to arrange training dates even between two fairly local practices.<br>Practice staff wished to see a live demonstration of the template, which was impossible away from the practice.	In house training per practice. Could give a live demonstration of the template and discuss procedures specific to practice organisation and requirements.
Eligible patients based on all chronic conditions included in the Quality and Outcomes Framework.	GPs expressed concern that not all patients warranted extra time as did not all have significant morbidity. Some patients concurred.	Review of eligibility criteria: Amalgamated chronic kidney disease with the cardiovascular group due to similar management. Removed osteoporosis.
Replacement of single condition clinics by 3D review clinics.	Some practices did not always cancel existing clinic reviews, leading to duplication. Some patients continued to book appointments e.g. for regular blood tests that they expected.	Create a checklist of changes that are required by the practice. Discussed at a post-training meeting (with lead administrator and/or practice manager). Obtained extra funding to reimburse time for rearranging appointment recall systems.
Longer appointments offered with usual GP between reviews	Practices concerned with committing longer appointments between reviews. 3D card creating an expectation amongst patients who may not need longer appointments	Wording changed on 3D card. Practices commit only to allow possibility of longer appointments when appropriate. Specific training of receptionists to suggest scripts for arranging longer appointments with usual GP.
Expect nurse to do first 3D	Different levels of experience	Allow a certain level of flexibility

review appointment followed approximately one week later by a GP 3D review.	and training amongst nurses. Some chronic conditions nurses e.g. diabetes nurses already do medication reviews and care planning. Worried about de-skilling	dependent on local skills and experience. Suggest some cases could use a Health Care Assistant for tests and blood tests, then some nurses can do some of the GP aspects of the template. Patients with diabetes should see a diabetes-trained nurse for their 3D reviews.
Use of template to guide only relevant tests and questions.	Some GPs did not use the template. Requires time to get used to it to using effectively. Nurses unhappy about asking some of the questions on the template.	Created an aide memoire to remind clinicians of the key elements to include in each of the reviews. Revised training to include a live demonstration of the template. Some questions streamlined or moved from nurse template into GP template.
Patients should be given a print out of their agenda and personalised health plan.	Patients not sure how to answer questions about what is most important to them. Nurses and GPs unsure what to put in care planning sections Technical problems with printing agenda and health plan.	Practices provided with a template appointment letter asking patients to think about what affects their health and wellbeing, so that they are prepared for the review. Care planning reviewed in training. Technical issues resolved.
